# Association of Bioelectrical Impedance Phase Angle with Physical Performance and Nutrient Intake of Older Adults

**DOI:** 10.3390/nu15061458

**Published:** 2023-03-17

**Authors:** Sandra Unterberger, Rudolf Aschauer, Patrick A. Zöhrer, Agnes Draxler, Mirjam Aschauer, Benno Kager, Bernhard Franzke, Eva-Maria Strasser, Karl-Heinz Wagner, Barbara Wessner

**Affiliations:** 1 Research Platform Active Ageing, University of Vienna, Josef-Holaubek-Platz 2, 1090 Vienna, Austria; sandra.unterberger@univie.ac.at (S.U.); rudolf.aschauer@univie.ac.at (R.A.); patrick.zoehrer@univie.ac.at (P.A.Z.); bernhard.franzke@univie.ac.at (B.F.); karl-heinz.wagner@univie.ac.at (K.-H.W.); 2 Institute of Sport Science, Centre for Sport Science and University Sports, University of Vienna, Auf der Schmelz 6, 1150 Vienna, Austria; mirjam.aschauer@gmx.at (M.A.); benno.kager@gmail.com (B.K.); 3 Vienna Doctoral School of Pharmaceutical, Nutritional and Sport Sciences (PhaNuSpo), University of Vienna, Josef-Holaubek-Platz 2, 1090 Vienna, Austria; agnes.draxler@univie.ac.at; 4 Department of Nutritional Sciences, Faculty of Life Sciences, University of Vienna, Josef-Holaubek-Platz 2, 1090 Vienna, Austria; 5 Karl Landsteiner Institute for Remobilization and Functional Health/Institute for Physical Medicine and Rehabilitation, Kaiser Franz Joseph Hospital, Social Medical Center South, 1100 Vienna, Austria; eva-maria.strasser@gesundheitsverbund.at

**Keywords:** bioelectrical impedance analysis, phase angle, physical function, elderly

## Abstract

In recent years, the phase angle (PhA) as a raw bioelectrical impedance analysis variable has gained attention to assess cell integrity and its association to physical performance in either sports-related or clinical settings. However, data on healthy older adults are scarce. Therefore, data on body composition, physical performance and macronutrient intake from older adults (n = 326, 59.2% women, 75.2 ± 7.2 years) were retrospectively analyzed. Physical performance was evaluated by the Senior Fitness Test battery, gait speed, timed up and go and handgrip strength. Body composition was determined by the BIA and dual-energy X-ray absorptiometry (from a subgroup of n = 51). The PhA was negatively associated with the timed up and go test and age (r = −0.312 and −0.537, *p* < 0.001), and positively associated with the 6 min walk test, 30 s chair stand, handgrip strength, gait speed and physical performance score (r = 0.170–0.554, *p* < 0.05), but not protein intake (r = 0.050, *p* = 0.386). Hierarchical multiple regression analysis showed that especially age, sex, BMI, but also the PhA predicted the performance test outcomes. In conclusion, the PhA seems to be an interesting contributor to physical performance, but sex- and age-specific norm values still need to be determined.

## 1. Introduction

Body composition plays a significant role in determining overall health, fitness and nutritional status. It is closely associated with various diseases, including cardiovascular diseases, diabetes, osteoporosis and various types of cancer [1,2,3]. As we age, physiological changes occur that impact body composition by lowering muscle mass and increasing body fat, whereby especially fat accumulation within muscles has been shown to be related to muscle weakness and poor function [4]. While sarcopenia is defined by the European Working Group on Sarcopenia in Older People as the loss of muscle mass, strength and function with ageing [5], frailty is a more general concept that goes beyond sarcopenia by including a range of physical, cognitive and social factors [6].

Several clinical scales and assessment methods exist for the diagnosis of sarcopenia and frailty [7]. Thereby, the assessment of physical function can be time-consuming or difficult, especially in the case of frailty, which is often complicated by comorbidities. In contrast, body composition is routinely determined by various methods [8] and, although debated, dual-energy X-ray absorptiometry (DXA) is widely considered as a gold standard for body composition assessment [9]. However, bioelectrical impedance analysis (BIA) is a promising non-invasive tool that can reliably estimate body fat mass, muscle mass, and hydration status in various clinical and non-clinical populations [10,11,12]. It has to be mentioned that BIA and DXA are based on different theoretical models that categorize the body into various compartments, and the interchangeable use of terms to describe these components often leads to confusion and misunderstandings when comparing them. The DXA method breaks down body composition into three components: fat mass, lean soft tissue and bone mineral content, while the BIA method provides only a two-component analysis of body composition, divided into lean body mass and fat mass. To compare these two methods, it is important to consider the difference in fat-free mass and compare the sum of lean soft tissue and bone mineral content for DXA with the lean body mass for BIA [13].

Most interestingly, a direct measure derived from the BIA, the phase angle (PhA), reflects the resistance and reactance of the body in response to the application of an external current. It is dependent on lean body mass and hydration status and, therefore, a decrease in muscle mass tends to decrease the PhA. The PhA can be quickly assessed, even at the bedside, and contrary to other BIA markers it is measured directly, avoiding errors attributable to regression equations [14]. Moreover, it has been shown that the PhA is associated with sarcopenia [15] and frailty [16,17], but data on the associations between raw BIA values and physical performance in an ageing population are scarce and inconclusive [18,19].

Furthermore, it has been shown that the PhA might be a useful screening tool to assess nutritional risk [20]. Interestingly, also nutritional behavior, such as the adherence to a Mediterranean diet [21] or higher dietary quality [22], was associated with the PhA in healthy populations. A very recent study suggests using the PhA assessment as a diagnostic tool to detect early changes in inflammatory parameters in response to a very low-calorie ketogenic diet [23]. It has been shown that protein intake is associated with muscle mass [24], but there is no information on whether protein (or macronutrient) intake is related to the PhA.

Therefore, we aimed (a) to describe and validate sex-specific raw BIA values in an older population with DXA parameters, and (b) to determine the association between the PhA and physical performance, also taking macronutrient intake into consideration.

## 2. Materials and Methods

This study is a secondary analysis of baseline data from three different randomized controlled trials, the Vienna Active Ageing Study [25], the NutriAging Protein Study [26] and the NutriAging Vitamin D Study [27], from which BIA and performance data are derived and which will be described in further detail in the next section.

### 2.1. Study Designs and Population Characteristics

#### 2.1.1. Vienna Active Ageing Study

The Vienna Active Ageing Study (VAAS) was conducted between 2011 and 2013 as a randomized controlled intervention study, with the aim being to investigate whether progressive resistance training with elastic bands over six weeks would influence physical performance in combination with and without nutrient supplementation. The trial was authorized by the Ethics Committee of the City of Vienna (EK-11-151-0811) and registered at ClinicalTrials.gov (NCT01775111) [25].

Participants comprised untrained, institutionalized older men and women over 65 years of age without severe health problems. The exclusion criteria were cognitive impairment (Mini-Mental State Examination score, MMSE < 23), chronic diseases that would not have allowed participation in sporting activities, serious cardiovascular diseases, diabetic retinopathy and regular use of cortisone-containing drugs.

**Figure 1:**
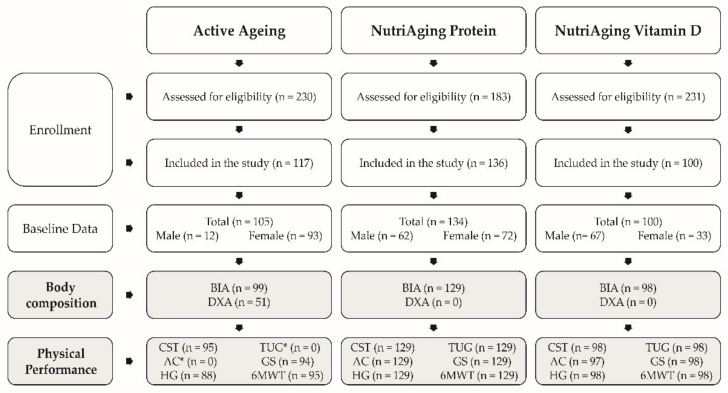
Participants’ flow. RT = resistance training, CST = 30 s chair stand, AC = 30 s arm curl, HG = handgrip strength, TUG = timed up and go test, GS = gait speed, 6MWT = 6 min walk test, * not measured in the Active Ageing Study.

Baseline BIA data were available from 99 participants. Physical performance parameters included data from 30-s chair stand test and 6-min walk test (n = 95), handgrip strength (n = 88) and gait speed (n = 94). The 30-s arm curl and the timed up and go test were not assessed in this study (Figure 1).

#### 2.1.2. NutriAging Protein Study

The NutriAging Protein Study used a randomized, controlled, observer-blind trial design to investigate the impact of protein intake, with or without resistance training, on physical performance. The study population was randomly divided into the following groups: control group (observation only), recommended or high-protein intake plus resistance training groups. After the initial assessment, there were two phases, a six week nutritional observation/counselling phase, followed by an eight week phase in which resistance training was added to the nutritional intervention. Data were collected at the baseline, after 8 and 17 weeks. The trial was been approved by the Ethics Committee of the University of Vienna (00322) and registered at ClinicalTrials.gov (NCT04023513). The study duration was from July to December 2018 [26].

Included subjects were untrained community-dwelling women and men between 65 and 85 years. The following criteria led to exclusion: cognitive impairment (MMSE < 23), acute and chronic illnesses prohibiting resistance training, severe cardiovascular disease, diabetic retinopathy, manifest osteoporosis, anticoagulant or cortisone medications, a frailty index ≥ 3, or the need for walking aids.

The BIA data were available for 129 persons and all physical performance parameters were also available for these participants (Figure 1).

#### 2.1.3. NutriAging Vitamin D Study

This trial was designed as a randomized, placebo-controlled, double-blind trial and examined the effect of vitamin D3 supplementation, with and without resistance training, on physical function. Participants were randomly assigned to a control group, a vitamin D3 daily group, or vitamin D3 monthly group. This study was also divided into two phases. The first phase included vitamin D3 or placebo supplementation, followed by the second phase, which included supplementation extended with resistance training. At the baseline, after the first phase and after the second phase (supplementation plus resistance training), physical performance parameters were collected. The Ethics Committee of the University of Vienna approved the study protocol (00390). The study was registered at ClinicalTrials.gov (NCT04341818) and was conducted from February to July 2019 [27].

The inclusion criteria for the participants were as follows: women or men between the ages of 65 and 85 years, with an independent lifestyle and no cognitive impairment (MMSE > 23). Individuals were excluded from the study if they had: a 25(OH)D level >30 ng/mL before study entry, the need for walking aids, chronic medical conditions that contraindicate resistance training, serious cardiovascular disease, diabetic retinopathy, osteoporosis or osteopenia with vitamin D and/or calcium supplementation, renal disease, renal stones, parathyroid hormone level disorder, cardiac glycoside use, diuretic (thiazide) use, calcium level disorder, a frailty index ≥ 3, regular intake of cortisone or antibiotics (in the last six months) and regular resistance training (>1x/week) in the last six months before study entry.

For 98 participants, the BIA data were available. Apart from the 30 s arm curl test (n = 97), all physical performance measures were available for the study population (Figure 1).

#### 2.1.4. Participants’ Flow

A total of 353 subjects, from all three studies, were included in these analyses. BIA data was not available for 27 people who were, therefore, excluded from the analyses, which finally included data from 326 subjects (Figure 1).

### 2.2. Outcomes

For this secondary analysis, the PhA was considered as a primary outcome. Further outcomes were BIA resistance and reactance, as well as body composition estimates and physical performance tests, such as a 30 s chair stand, handgrip strength, 30 s arm curl test, timed up and go, gait speed and 6 min walk test.

### 2.3. Body Composition

Body mass was measured to the nearest 0.1 kg and standing height to the nearest 0.1 cm in light clothes. The BMI (kg/m²) was calculated from body mass (kg) and height (m) measurements.

A multifrequency (5, 50, 100 kHz) BIA system (Nutriguard-MS, Data Input, GmbH, Germany) was used to measure the PhA, resistance and reactance (standard placement of surface electrodes). To avoid any bias due to diet, all participants were evaluated in the morning after an overnight fast. A more detailed description is provided in the respective original studies from our research group [25,26,27].

However, resistance, reactance and the resultant PhA values were only derived from data using a frequency of 50 kHz, while estimates for lean body mass (extracellular plus body cell mass), total body water and body fat were derived from the multifrequency device software (NutriPlus software, Version 5.1, Data Input, GmbH, Germany). In addition, skeletal muscle mass (SM) was calculated using a population specific Equation (1):

SM (kg) = [(Ht²/R * 0.401) + (sex * 3.825) + (age * 0.071)] + 5.102(1)

where Ht is height in cm, R is resistance in Ohm, sex (1 = for men and 0 = for women) and age is indicated in years [28].

For a subset of the population, whole body scans were performed using dual-energy X-ray absorptiometry (DXA, Hologic^®^, Hologic Inc., Bedford, MA, USA). Total and segmental fat-free mass (FFM, lean soft tissue plus bone mineral content) and fat mass (FM) were measured based on their X-ray attenuation properties [29].

To compare the FFM measured by the BIA and DXA, the lean body mass (including the extracellular and body cell mass) was used as the estimate for the BIA, while the estimate for the DXA was lean soft tissue plus bone mineral content.

### 2.4. Physical Performance

Physical performance was assessed by the 30 s chair stand, 30 s arm curl and handgrip strength tests to measure the lower and upper extremity muscle strength, a 6 min walk test to assess aerobic endurance, as well as gait speed and timed up and go tests to measure agility and dynamic balance.

The results from the 30 s chair stand, handgrip strength, gait speed and 6 min walk tests were used to calculate the physical performance score based on the weighted sum method. After scaling the test results into sex-specific Z-scores, a principal component analysis was conducted to obtain the loading value for each test. The performance score of each subject was calculated by multiplying the Z-scores by the corresponding loading value before summation [30].

### 2.5. Assessment of Nutrient Intake

To assess the macronutrient intake, the participants’ nutritional data were recorded via 24 h dietary recalls in a personal interview. The dietary intake data were recorded and analyzed using GloboDiet^®^ software. The total energy intake (kcal), as well as the absolute (g/day) and relative per body weight (g/kg BW/d) intake of protein, carbohydrates and fat were used in this study [31].

### 2.6. Assessment of Comorbidities

During the medical examination, the following comorbidities were assessed: hyperlipidaemia, type 2 diabetes mellitus, osteoporosis, and a history of heart disease and cancer. According to World Health Organization criteria, obesity was defined as BMI ≥ 30 kg/m² and hypertension as systolic and diastolic blood pressure above 140/90 mm Hg [32].

### 2.7. Statistical Analysis

Statistical analyses were performed using IBM SPSS Statistics 27 (IBM Corporation, Armonk, New York, NY, USA) and R software (v4.1.1, R Foundation for Statistical Computing, Vienna, Austria). Continuous variables were reported as mean ± standard deviation and categorical variables were presented as frequencies and percentages. The independent samples *t*-test and chi-square test were used to compare the sex differences. The relationships between the variables were determined using the Pearson’s product-moment coefficient (r). According to Pearson’s coefficient, the correlations were graded as weak (r < 0.3), moderate (r = 0.3–0.5), or strong (r > 0.5) [33]. Correlation matrix visualizations were conducted using the corrplot package in R. Bland–Altman plots were created to further examine the agreement between the BIA and DXA. Hierarchical multiple regression analysis was conducted using the 30 s chair stand, handgrip strength, 30 s arm curl, timed up and go, gait speed, 6 min walk test and physical performance score as dependent variables. Age, sex (female = 0, male = 1) and BMI were previously selected for the first regression model, based on their effects on performance, as described in the literature. The independent variables were inserted into the different regression models: model 1 consisted of age, sex and BMI (forced entry); model 2 and model 3 were derived from model 1 and the stepwise inserted variables resistance, reactance and/or PhA. All assumptions, error independence (Durbin–Watson values between 1–3), non-multicollinearity (tolerance values > 0.1; VIF values < 10), homoscedasticity (standardized residual values between −3 and +3) and non-influential cases (Cook’s distance values < 1) for multiple linear regression were tested for all models. *p*-values less than 0.050 were considered as significant.

## 3. Results

### 3.1. Participants’ Characteristics

The participants were 193 females and 133 males with a mean age of 75.2 ± 7.2 years. Compared to the male group, the female group was older, had lower weight, height, waist, arm and calf circumference. On the other hand, the male group had a smaller waist circumference and lower prevalence of obesity, hyperlipidemia and osteoporosis, when compared with the female group. Across the study, 47.5% persons were overweight and 25.2% were obese. As summarized in Table 1, women and men were comparable for BMI and incidence of hypertension, type 2 diabetes mellitus, and a history of heart disease and cancer. Absolute energy and macronutrient intake was higher in men than in women, although no relative difference in dietary intake was observed, except for fat intake.

The BIA and physical performance parameters of the total study population, as well as the differences between the male and female participants, are shown in Table 2. For the raw values of the BIA, it was observed that men showed higher values for the PhA compared to women, while women had higher resistance and reactance (*p* < 0.001) values. Females were characterized by lower total body water, lean body mass, extracellular mass, body cell mass and SM and by higher body fat mass/percentage, as compared to males (*p* < 0.001).

The physical performance parameters also showed differences between the male and female participants, except for the sex-specific physical performance score. Men completed more repetitions in the 30 s chair stand and 30 s arm curl, achieved higher values in handgrip strength, walking speed, distance covered in the 6 min walk test and were faster at the timed up and go in comparison to women (*p* < 0.001).

### 3.2. Agreement of Body Composition Parameters Measured by BIA and DXA

**Figure 2:**
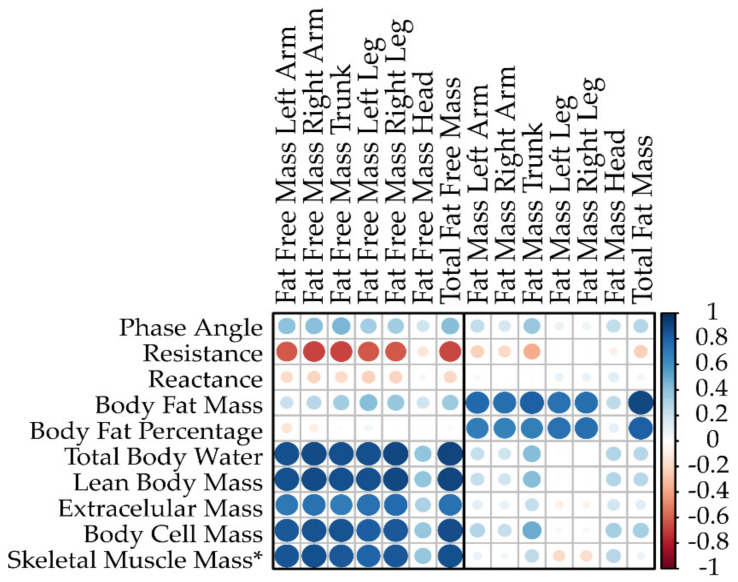
Correlation matrix for the BIA and DXA parameters: the red and blue dots correspond to negative and positive correlations, respectively. Small dots with light colors represent lower intensity correlations, and larger dots with darker colors correspond to higher intensity correlations. * As estimated by Janssen et al. (2000) [28].

In a subsample of 51 participants (46 females and 5 males) from the Vienna Active Ageing Study, body composition parameters were compared for agreement between the BIA and DXA. Pearson correlations for the BIA and DXA parameters are graphically displayed in Figure 2, whereas the detailed correlation coefficients and significance levels are shown in Table 3.

The FFM (DXA) showed strong positive correlations with the device-derived BIA parameters: lean body mass (r = 0.871–0.917, *p* < 0.001), total body water (r = 0.871–0.917, *p* < 0.001), extracellular mass (r = 0.690–0.778, *p* < 0.001) and body cell mass (r = 0.824–0.890, *p* < 0.001). Body fat mass correlated weakly to moderately and positively with the FFM (r = 0.276–0.422 *p* < 0.050), while body fat percentage did not.

In addition, the PhA showed significant moderate positive correlations with the FFM parameters: total FFM (r = 0.425, *p* = 0.002), FFM arm (left: r = 0. 408, *p* = 0.003; right: r = 0.415, *p* = 0.002), FFM trunk (r = 0.453, *p* = 0.001) and FFM leg (left: r = 0.344, *p* = 0.013; right: r = 0.350, *p* = 0.012). The FFM head was not associated with the PhA (*p* = 0.198). In contrast to the PhA, resistance showed strong negative correlation with the total FFM (r = −0.665, *p* < 0.001) and segment specific FFM (r = −0.613 to −0.680, *p* < 0.001).

The total FM (DXA), body fat mass and percentage (BIA) demonstrated significant strong positive correlations, r = 0.907 and r = 0.816, *p* < 0.001, respectively. However, there were no significant correlations between the total FM and the device-derived BIA parameters (all *p* > 0.050), with the exception of body cell mass (r = 0.336, *p* = 0.016).

When using a population-specific equation to determine skeletal muscle mass, similar strong correlations were found in comparison to device-derived body cell mass: total FFM (r = 0.877, *p* < 0.001), FFM arms (left: r = 0.852, *p* < 0.001; right: r = 0.877, *p* < 0.001), FFM trunk (r = 0.842, *p* < 0.001) and FFM legs (left: r = 0.802, *p* < 0.001; right: r = 0.845, *p* < 0.001).

**Figure 3:**
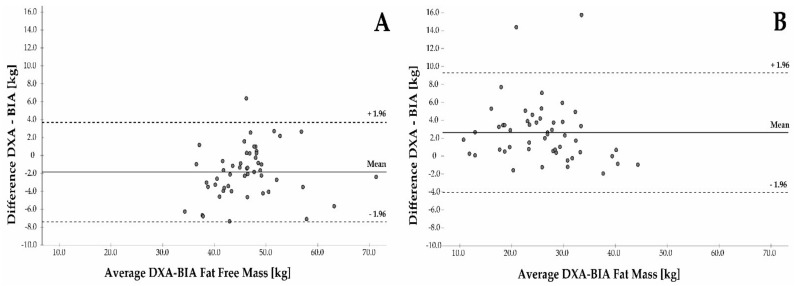
Bland−Altman analysis for the fat-free mass (FFM (**A**)) measured and for the fat mass (FM (**B**)) measured by the BIA and DXA.

Additionally, the agreement between the two methods was evaluated using Bland–Altman plots (Figure 3). The BIA results displayed good absolute agreement with the DXA for the assessment of either the FFM or FM. There was a significant mean difference between the FFM and lean body mass of −1.9 kg (95% CI −2.65, −1.06 kg). The difference between the FM and body fat mass accounted for +2.6 kg (95% CI 1.67, 3.59 kg). The limits of agreement were narrower for the FFM (−7.4 to +3.7 kg) as compared to the FM (−4.0 to +9.3 kg). The BIA tended to overestimate the FFM and to underestimate the FM in comparison to DXA.

### 3.3. Association between BIA Raw Parameters, Physical Performance, Age, BMI and Nutrient Intake

**Figure 4:**
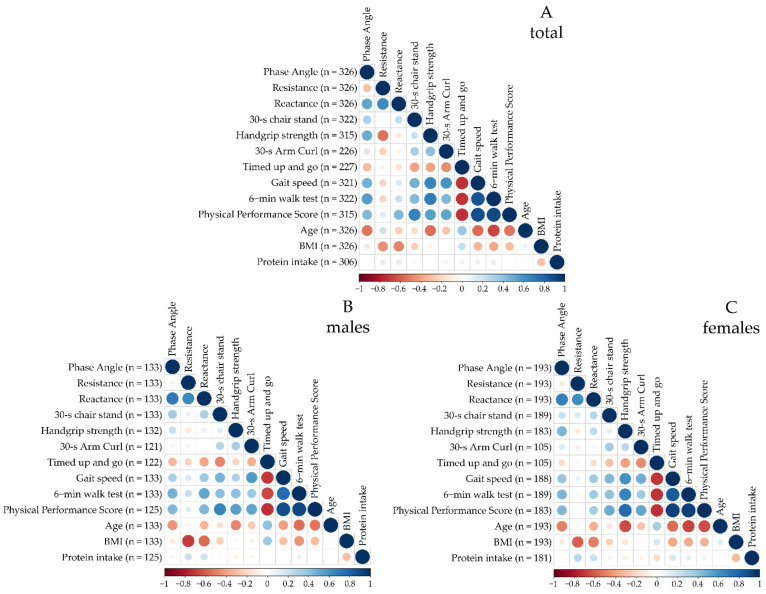
Correlation matrix for the total population (**A**), males (**B**) and females (**C**). The red and blue dots correspond to negative and positive correlations, respectively. Small dots with light colors represent lower intensity correlations, and larger dots with darker colors correspond to higher intensity correlations.

In order to determine the relationships between the raw BIA parameters, physical function and other influencing factors, such as age, BMI and nutrient intake, a Pearson’s product-moment correlation matrix was created (Figure 4, Table 4). Across the total population, the PhA was negatively correlated with resistance (r = −0.293, *p* < 0.001) and positively correlated with reactance (r = 0.524, *p* < 0.001). A strong positive correlation was found between the PhA and the 6 min walk test (r = 0.554, *p* < 0.001), but a negative correlation was found between the PhA and age (r = −0.537, *p* < 0.001). The PhA moderately correlated with the 30 s chair stand (r = 0.302, *p* < 0.001), handgrip strength (r = 0.488, *p* < 0.001), gait speed (r = 0.470, *p* < 0.001), physical performance score (r = 0.408, *p* < 0.001) and timed up and go (r = −0.312, *p* < 0.001). Weak correlations were observed between the PhA and 30 s arm curl (r = 0.170, *p* = 0.011) and BMI (r = −0.122, *p* = 0.028), whereas protein intake did not correlate with the PhA (*p* > 0.050).

Male population: A strong positive relationship was found for the phase angle and reactance (r = 0.700, *p* < 0.001). Age (r = −0.450, *p* < 0.001) and timed up and go (r = −0.324, *p* < 0.001) showed moderate negative correlations, while the 30 s chair stand (r = 0.344, *p* < 0.001), gait speed (r = 0.317, *p* < 0.001), 6 min walk test (r = 0.482, *p* < 0.001) and physical performance score (r = 0.443, *p* < 0.001) were positively correlated.

Female population: For the female group, similar results were found. The phase angle had a strong positive correlation with resistance (r = 0.678, *p* < 0.001) and a negative correlation with age (r = −0.507, *p* < 0.001). Moderate associations were found with the handgrip strength (r = 0.453, *p* < 0.001), gait speed (r = 0.379, *p* < 0.001), 6 min walk test (r = 0.449, *p* < 0.001) and physical performance score (r = 0.442, *p* < 0.001). A weak correlation was reported between the phase angle and 30 s chair stand (r = 0.217, *p* = 0.003). Furthermore, in contrast to men, there was no significant correlation with the timed up and go among women (*p* > 0.050). The resistance, 30 s arm curl, BMI and protein intake showed no significant association with the phase angle for women and men (*p* > 0.050).

### 3.4. Hierarchical Multiple Regression Analyses

Due to the absence of a significant correlation between the nutrient intake parameters and functional tests, they were not included in the multiple regression analyses. Hierarchical multiple regression analyses were performed to examine the impact on physical performance by entering independent variables as blocks into the models. Including sex, age and BMI as the first block significantly explained: 10.8% of the 30 s chair stand, 77.5% of the handgrip strength, 13.6% of 30 s arm curl, 30.7% of the timed up and go, 57.5% of the gait speed, 68.2% of 6 min walk test and 47.1% of the physical performance score.

As the second block, resistance, reactance and/or the PhA were stepwise included in further models. The addition of the PhA to the multiple regression model was statistically significant in the 30 s chair stand and resulted in an increase in R^2^ from 0.108 to 0.142, F(4, 306) = 12.607, *p* < 0.001. When the PhA was added to the handgrip strength, the R^2^ increased significantly to 0.780 F(5, 305) = 220.890, *p* < 0.001. In the timed up and go, the addition of the PhA led to a significant increase in the R^2^ to 0.320, F(4, 220) = 25.868, *p* < 0.001. The gait speed also showed a significant increase in the R^2^ of 0.585, F(4, 312) = 109.820, *p* < 0.001, when the PhA was added to the regression model. The addition of the PhA in the physical performance score increased the R^2^ to 0.500, F(4, 309) = 77.334, *p* < 0.001. Adding the PhA in the 6 min walk test resulted in a significant increase in the R^2^ of 0.701, F(4, 312) = 183.220, *p* < 0.001 and adding resistance increased the R^2^ to 0.714, F(5, 311) = 155.229, *p* < 0.001. With the addition of resistance in the 30 s arm curl resulted in a significant increase in the R^2^ of 0.155, F(4, 220) = 10.110, *p* < 0.001. Adding resistance in the handgrip strength resulted in a statistically significant increase in the R^2^ of 0.778, F(4, 306) = 272.191, *p* < 0.001. Including reactance in the physical performance score led to a significant increase in the R^2^ to 0.509, F(5, 308) = 63.850, *p* < 0.001 (Table 5).

The analysis resulted in the following equations for the physical performance tests and score (Equations (2)–(7)), where age is indicated in years, sex (1 = for men and 0 = for women), BMI is body mass index in kg/m^2^, PhA is phase angle in degree, R is resistance in ohm and Xc is reactance in ohm.

30 s chair stand (reps) = [(age * −0.016) + (sex * 0.351) + (BMI * −0.132) + (PhA * 0.885)] + 12.827(2)

Handgrip strength (kg) = [(age * −0.508) + (sex * 13.702) + (BMI * −0.138) + (R * −0.015) + (PhA * 1.031)] + 69.060(3)

Timed up and go (s) = [(age * 0.071) + (sex * −0.597) + (BMI * 0.065) + (PhA * −0.196)] + −0.122(4)

Gait speed (s) = [(age * −0.036) + (sex * 0.358) + (BMI * −0.028) + (PhA * 0.091)] + 44.851(5)

6 min walk test (m) = [(age * −9.849) + (sex * 40.679) + (BMI * −11.397) + (PhA * 57.426) + (R * −3.704)] + 1443.476(6)

PPscore (-) = [(age * −0.187) + (sex * −1.687) + (BMI * −0.196) + (PhA * 1.076) + (Xc * −0.054] + 17.116(7)

The PhA did not contribute significantly to the regression model for the 30 s arm curl, but resistance did, which led to the following Equation (8):

30 s arm curl (reps) = [(age * −0.200) + (sex * 0.993) + (BMI * −0.151) + (R * −0.012)] + 40.573(8)

## 4. Discussion

The aim of this study was to describe the sex-specific raw BIA parameters in an older population and to validate the agreement between the BIA and DXA parameters, as well as assess the associations between the PhA, physical performance and macronutrient intake.

In agreement with the literature, the PhA of males was higher as compared to females, as a result of the lower reactance and resistance values of males [18,34,35,36]. It is known that the higher FFM and fluid volume of males are associated with a decrease in resistance, whereas their lower body fat mass results in lower reactance values [35]. When comparing the BIA-derived parameters to DXA, which is considered as a gold standard for measuring body composition [13], the PhA correlated moderately with the FFM and weakly with the FM. Interestingly, resistance correlated even stronger with the FFM, while reactance was neither correlated with the FM nor with the FFM. In addition to examining the relationship between the PhA, FFM and FM, the agreement between the BIA and DXA measurements for the FFM and FM was assessed using Bland–Altman plots. Our results showed that the BIA overestimated the FFM and underestimated the FM compared with DXA, which is in line with studies on middle-aged persons [37,38] and COPD patients [39].

For the practical application of the PhA, reference values and cut-off points are needed to assess the individual deviations from the population-based average. The reference values for healthy older adults (BMI of 19–25 kg/m²) range from 4.7 to 6.4° for women and 4.7 to 6.6° for men [34]. In our study, the average PhA value for women was at the lower end of the reference range, while that for men was in the lower third. This could be due to the slightly higher BMI in both sexes [40,41,42]. However, due to different sample characteristics and the variety of devices being used, various cut-off values are under discussion [18,34]. In a study of older intensive care patients, the cut-off value for low PhA was set at <4.8° [43]. For community-dwelling older persons with increased risk of incident disability, the cut-off value was set to ≤4.95° for men and ≤4.35° for women [44]. In another study, low PhA values of ≤4.1° were found to be determinants of frailty and sarcopenia in older adults [16].

Lower PhA values may indicate decreased cell integrity or cell death, whereas higher values can be attributed to greater cellularity (higher body cell mass relative to FFM), cellular integrity and cellular function [40,41]. Consequently, the PhA might reflect not only muscle mass, but also the muscles’ functional quality, explaining the relationship between the PhA and physical performance. This study confirmed the associations between the PhA and the 6 min walk test, 30 s chair stand, 30 s arm curl, timed up and go, gait speed, handgrip strength and physical performance score. These findings support the suggestion that the PhA could be a useful biomarker to estimate physical performance [18,45,46]. Besides functional performance, the PhA has been shown to correlate with muscle strength or power [47,48], knee extensor strength [49] and maximal torque of plantar and dorsal flexion [50], in middle-aged or older populations [51].

In order to prove whether the raw BIA parameters, the PhA and resistance and reactance, contribute to the prediction of physical performance, multiple regressions were computed. The PhA was identified as a predictor of the 6 min walk test, gait speed, timed up and go, 30 s chair stand, handgrip strength and physical performance score, whereas it did not contribute to the prediction of the 30 s arm curl test. It could be argued that there is a difference between the lower and upper body function, as muscle mass also differs between the lower and upper body [52].

Interestingly, age, sex and BMI with or without the addition of the PhA, resistance or reactance, explained a relatively high amount of the variability in the handgrip strength, gait speed and 6 min walk test, which was visibly lower for the 30 s chair stand, 30 s arm curl and timed up and go tests. A study conducted on elderly Koreans investigated the ability to predict functional test outcomes using independent variables, such as sex, age, BMI and body fat percentage instead of PhA. The results of the study showed similar coefficients of determination for hand grip strength (R² = 0.773) and timed up and go (R² = 0.384), while the value for the 30 s chair stand test (R² = 0.296) was higher as compared to our developed equations [53]. One possible reason for higher values in the linear regression model could be that a higher body fat percentage can limit mobility and flexibility, making it more difficult to stand up from a seated position. Furthermore, repeated standing up and sitting down during the 30 s chair stand test is biomechanically more demanding and requires more torque and range of motion in the lower limbs than walking [54]. This means that standing up and sitting down requires coordination of the trunk and lower limb movements and control of balance and stability, in addition to the muscular strength associated with the PhA [55,56]. Furthermore, this could also apply to the 30 s arm curl test, as it requires a high degree of coordination compared to handgrip strength. Although further validation studies may be required, predicting and not measuring physical performance could be useful in settings where it is difficult or time-consuming to conduct a variety of functional performance tests, and the inclusion of raw BIA values could improve the predictions.

In addition to physical function, nutritional patterns are also an important factor in the health status of older people. However, in our study, no correlations were found between the PhA and the macronutrient intake parameter. The consensus statement from the European Society for Clinical Nutrition and Metabolism (ESPEN) recommends BMI to characterise malnutrition [57]. It has been shown that the standardized PhA is considerably lower in surgical patients [58] or advanced colorectal cancer patients [59] with impaired nutritional status, but the high proportion of overweight and obese participants, probably without severe malnutrition, could have masked this association.

Regardless of the encouraging results, the limitations to this study must be considered. We have analysed the PhA at a single frequency (50 kHz) in this study. A multi-frequency BIA measurement allows for more accurate measurement and differentiation of the lean body mass, total body water, intracellular water and extracellular water, based on their different tissue penetration than a single frequency measurement [60]. Therefore, it would be interesting to measure/analyze the PhA at different frequencies in future studies to gain a more comprehensive understanding of the prognostic utility of the PhA. This approach could potentially contribute to a more accurate estimate of the PhA. In further studies or secondary analyses, it would be interesting to include persons at risk of being underweight and/or sarcopenia to obtain a more precise understanding of the impact of the phase angle in relation to both physical performance and nutritional status. Nevertheless, we suggest the PhA as an interesting parameter as it can provide information about body composition and cell integrity, both factors of which are important for muscle function.

## 5. Conclusions

Higher PhA values are associated with better physical performance but are unrelated to macronutrient intake. The PhA seems to be an interesting parameter in the context of physical performance, as it is independent from the process of finding a suitable regression equation and might be useful for a broad range of populations and settings. In addition, the aspect of cell integrity is of particular interest, as the muscle cell and its contractile properties play an essential role in the context of physical fitness. Factors that can affect muscle cell integrity and, thus, physical performance include a lack of physical activity, age, certain diseases and probably, poor nutrition. In this study frail participants were excluded, but the PhA could be useful in situations where physical performance tests cannot be conducted.

## Figures and Tables

**Table 1:** Anthropometric, macronutrient intake and comorbidity characteristics of the participants.

	Total	Female	Male
Sex (f/m), (%)	326 (100%)	193 (59.2%)	133 (40.8%)
Study origin (Study 1/Study 2/Study 3), (%)	99 (30.4%)/ 119 (39.6%)/ 98 (30.1%)	88 (45.6%)/ 72 (37.3%)/ 33 (17.1%)	11 (8.3%)/ 57 (42.9%)/ 65 (48.9%)
Age (years)	75.2 ± 7.2	77.0 ± 7.2	72.7 ± 6.4
Body mass (kg)	76.2 ± 14.8	70.7 ±13.0	84.3 ± 13.4
Height (m)	1.7 ± 0.1	1.6 ± 0.1	1.8 ± 0.1
Body mass index (kg/m²)	27.5 ± 4.7	27.8 ± 5.0	27.1 ± 4.2
BMI categories (<25.0 kg/m², 25.0–29.9 kg/m², ≥ 30.0 kg/m²) (n, %)	89 (27.3%)/ 155 (47.5%)/ 82 (25.2%)	54 (28.0%)/ 80 (41.4%)/ 59 (30.6%)	35 (26.3%)/ 75 (56.4%)/ 23 (17.3%)
Waist circumference (cm), n = 315	94.7 ± 12.2	90.6 ± 11.3	100.4 ± 11.1
Hip circumference (cm), n = 315	105.1 ± 9.6	106.0 ± 10.5	104.0 ± 8.1
Waist to hip ratio (-), n = 315	0.9 ± 0.1	0.9 ± 0.1	1.0 ± 0.1
Arm circumference right (cm), n = 310	30.3 ± 3.4	29.8 ± 3.5	31.0 ± 3.2
Calf circumference right (cm), n = 310	37.1 ± 3.2	36.5 ± 3.2	37.9 ± 3.0
Energy intake (kcal), n = 306	1748.9 ± 647.4	1552.7 ± 503.1	2032.9 ± 725.2
Energy intake (kcal/kg BW), n = 306	23.4 ± 8.8	22.0 ± 8.6	24.5 ± 9.0
Protein intake (g/day), n = 306	61.9 ± 25.8	55.6 ± 22.6	71.0 ± 27.4
Protein intake (g/kg BW/day), n = 306	0.83 ± 0.36	0.81 ± 0.38	0.85 ± 0.33
Carbohydrate intake (g/day), n = 306	183.6 ± 75.2	168.5 ± 63.8	205.5 ± 84.7
Carbohydrate intake (g/kg BW/day), n = 306	2.48 ± 1.09	2.47 ± 1.08	2.49 ± 1.11
Fat intake (g/day), n = 306	72.2 ± 36.1	62.5 ± 27.1	86.4 ± 42.5
Fat intake (g/kg BW/day), n = 306	0.96 ± 0.46	0.91 ± 0.43	1.04 ± 0.51
**Comorbidities, n = 326**			
Hypertension (number (% of total))	178 (54.6%)	109 (56.5%)	69 (51.9%)
Hyperlipidemia (number (% of total))	55 (16.9%)	42 (21.8%)	12 (9.0%)
Diabetes mellitus type 2 (number (% of total))	33 (10.1%)	21 (10.9%)	12 (9.0%)
History of cardiac diseases (number (% of total))	38 (11.7%)	24 (12.4%)	14 (10.5%)
Osteoporosis (number (% of total))	48 (14.7%)	46 (23.8%)	2 (1.5%)
History of cancer (number (% of total))	34 (10.4%)	15 (7.8%)	19 (14.3%)

Notes: Values are shown as mean ± standard deviation or as absolute and relative frequencies.

**Table 2:** Bioelectrical impedance analysis and physical performance parameters.

	Total (n = 326)	Female (n = 193)	Male (n = 133)	*p*-Value	Effect Size
**Bioelectrical impedance parameters**					
Phase angle (°)	5.0 ± 0.7	4.7 ± 0.7	5.3 ± 0.7	<0.001	−0.813
Resistance (ohm)	495 ± 79	534 ± 68	438 ± 57	<0.001	1.512
Reactance (ohm)	43 ± 8	44 ± 7	40 ± 7	<0.001	0.525
Total body water (l)	40.6 ± 8.9	34.6 ± 4.0	49.5 ± 6.3	<0.001	−2.956
Lean body mass (kg)	55.6 ± 12.2	47.2 ± 5.5	67.7 ± 8.6	<0.001	−2.955
Extracellular mass (kg)	29.7 ± 6.2	26.0 ± 3.3	35.2 ± 5.4	<0.001	−2.156
Body cell mass (kg)	25.8 ± 6.8	21.2 ± 3.4	32.5 ± 4.8	<0.001	−2.829
Body fat mass (kg)	20.6 ± 8.9	23.3 ± 9.1	16.6 ± 6.7	<0.001	0.803
Body fat percentage (%)	26.8 ± 9.2	32.0 ± 7.4	19.2 ± 5.4	<0.001	1.904
Skeletal muscle mass (kg)	24.6 ± 7.6	19.1 ± 3.3	32.7 ± 4.2	<0.001	−3.665
**Physical performance parameters**					
Physical performance score (-), n = 315	0.06 ± 2.44	0.08 ± 2.52	0.04 ± 2.33	0.859	0.020
30 s chair stand (reps), n = 322	12.4 ± 3.6	11.9 ± 3.6	13.0 ± 3.5	0.009	−0.297
Handgrip strength (kg), n = 315	30.7 ± 11.2	23.2 ± 6.3	41.2 ± 7.4	<0.001	−2.655
30 s arm curl (reps), n = 226	17.1 ± 4.0	15.9 ± 3.5	18.1 ± 4.2	<0.001	−0.591
Timed up and go (s), n = 227	5.4 ± 1.1	5.8 ± 1.1	5.1 ± 1.1	<0.001	0.632
Gait speed (m/s), n = 321	2.0 ± 0.6	1.8 ± 0.5	2.4 ± 0.5	<0.001	−1.216
6 min walk test (m), n = 322	529.6 ± 141.4	471.2 ± 127.4	612.6 ± 117.0	<0.001	−1.147

Notes: Values are shown as mean ± standard deviation. *p*-values refer to differences between the groups (independent samples *t*-test). Effect size is given as Cohen’s d, whereby 0.200, 0.500 and 0.800 represent small, moderate or large effects, respectively.

**Table 3:** Correlation coefficients between the bioelectrical impedance analysis and DXA.

	Fat Free Mass						
	Arm Left	Arm Right	Trunk	Leg Left	Leg Right	Head	Total
Phase angle (°)	0.408 **	0.415 **	0.453 **	0.345 *	0.350 *	0.198	0.425 **
Resistance (ohm)	−0.613 ***	−0.671 ***	−0.680 ***	−0.617 ***	−0.610 ***	−0.132	−0.665 **
Reactance (ohm)	−0.182	−0.213	−0.188	−0.226	−0.219	0.041	−0.201
Total body water (l)	0.871 ***	0.898 ***	0.875 ***	0.877 ***	0.905 ***	0.393 **	0.917 **
Lean body mass (kg)	0.871 ***	0.898 ***	0.875 ***	0.877 ***	0.905 ***	0.392 **	0.917 **
Extracellular mass (kg)	0.715 ***	0.742 ***	0.690 ***	0.745 ***	0.778 **	0.316 *	0.750 **
Body cell mass (kg)	0.844 **	0.869 ***	0.869 ***	0.824 ***	0.849 ***	0.387 **	0.890 **
Body fat mass (kg)	0.214	0.276 *	0.344*	0.422 **	0.383 **	0.198	0.353 *
Body fat percentage (%)	−0.134	−0.090	−0.012	0.059	0.006	0.001	−0.024
Skeletal muscle mass (kg)	0.852 ***	0.877 ***	0.842 ***	0.802 ***	0.845 ***	0.388 **	0.877 **
	**Fat mass**						
	**Arm Left**	**Arm Right**	**Trunk**	**Leg Left**	**Leg Right**	**Head**	**Total**
Phase angle (°)	0.246	0.177	0.372 **	0.087	0.077	0.248	0.280 *
Resistance (ohm)	−0.223	−0.199	−0.367 **	−0.004	0.001	−0.078	−0.238
Reactance (ohm)	0.032	−0.002	0.017	0.088	0.083	0.126	0.052
Total body water (l)	0.237	0.198	0.423 **	−0.011	−0.001	0.296*	0.272
Lean body mass (kg)	0.236	0.197	0.422 **	−0.012	−0.003	0.295 *	0.271
Extracellular mass (kg)	0.113	0.099	0.234	−0.072	−0.062	0.188	0.121
Body cell mass (kg)	0.295 *	0.236	0.493 ***	0.033	0.036	0.334 *	0.336 *
Body fat mass (kg)	0.780 ***	0.756 ***	0.818 ***	0.741 ***	0.756 ***	0.252	0.907 ***
Body fat percentage (%)	0.691 ***	0.676 ***	0.684 ***	0.741 ***	0.755 ***	0.106	0.816 ***
Skeletal muscle mass (kg)	0.086	0.052	0.255	−0.187	−0.179	0.279*	0.078

Notes: * *p* < 0.050, ** *p* < 0.010, *** *p* < 0.001.

**Table 4:** Association between the BIA raw parameters, physical performance, age, BMI and nutrient intake.

		Phase Angle (°)	Resistance 50 kHz (ohm)	Reactance 50 kHz (Ohm)	30 s Chair Stand (WH)	Handgrip Strength Maximal (kg)	Arm Curl Test Maximal (WH)	Timed Up and Go (s)	Gait Speed (m/s)	6 min Walk Test (m)	Physical Performance Score	Age (Years)	Body Mass Index (kg/m²)
Resistance 50 kHz (ohm)	total	−0.293 ***											
	female	−0.106											
	male	−0.078											
Reactance 50 kHz (ohm)	total	0.524 ***	0.635 ***										
	female	0.678 ***	0.618 ***										
	male	0.700 ***	0.641 ***										
30 s chair stand (WH)	total	0.302 ***	0.022	0.251 ***									
	female	0.217 **	0.157 *	0.280 ***									
	male	0.344 ***	0.102	0.333 ***									
Handgrip strength maximal (kg)	total	0.488 ***	−0.542 ***	−0.114 *	0.175 **								
	female	0.453 ***	−0.120	0.251 **	0.089								
	male	0.241 **	−0.128	0.107	0.139								
Arm curl test maximal (WH)	total	0.170 *	−0.222 **	−0.079	0.333 ***	0.401 ***							
	female	0.154	−0.094	0.039	0.342 ***	0.266 **							
	male	0.060	−0.061	−0.008	0.305 **	0.318 ***							
Timed up and go (s)	total	−0.312 ***	0.053	−0.170 *	−0.433 ***	−0.387 ***	−0.501 ***						
	female	−0.148	−0.082	−0.176	−0.328 **	−0.363 ***	−0.603 ***						
	male	−0.324 ***	−0.234 ***	−0.392 ***	−0.488 ***	−0.197 *	−0.355 ***						
Gait speed (m/s)	total	0.470 ***	−0.218 ***	0.160 **	0.448 ***	0.656 ***	0.591 ***	−0.736 ***					
	female	0.379 ***	0.107	0.350 ***	0.426 ***	0.608 ***	0.496 ***	−0.717 ***					
	male	0.317 ***	0.169	0.361 ***	0.463 ***	0.314 ***	0.567 ***	−0.702 ***					
6 min walk test (m)	total	0.554 ***	−0.224 ***	0.231 ***	0.432 ***	0.679 ***	0.431 ***	−0.689 ***	0.870 ***				
	female	0.449 ***	0.034	0.357 ***	0.393 ***	0.654 ***	0.377 ***	−0.618 ***	0.841 ***				
	male	0.482 ***	0.222 *	0.533 ***	0.460 ***	0.405 ***	0.349 **	−0.676 ***	0.806 ***				
Physical performance score	total	0.408 ***	0.056	0.374 ***	0.558 ***	0.398 ***	0.372 ***	−0.539 ***	0.771 ***	0.796 ***			
	female	0.443 ***	0.018	0.346 ***	0.497 ***	0.777 ***	0.516 ***	−0.721 ***	0.923 ***	0.930 ***			
	male	0.443 ***	0.142	0.447 ***	0.670 ***	0.547 ***	0.526 ***	−0.726 ***	0.886 ***	0.908 ***			
Age (years)	total	−0.537 ***	0.214 ***	−0.248 ***	−0.168 **	−0.548 ***	−0.275 ***	0.378 ***	−0.565 ***	−0.668 ***	−0.577 ***		
	female	−0.507 ***	0.054	−0.353 ***	−0.103	−0.655 ***	−0.283 **	0.356 ***	−0.550 ***	−0.671 ***	−0.643 ***		
	male	−0.450 ***	0.054	−0.336 ***	−0.184 *	−0.468 ***	−0.247 **	0.377 ***	−0.452 ***	−0.571 ***	−0.535 ***		
Body mass index (kg/m²)	total	−0.122 *	−0.461 ***	−0.498 ***	−0.236 ***	−0.076	−0.005	0.235 ***	−0.337 ***	−0.391 ***	−0.332 ***	0.095	
	female	−0.107	−0.592 ***	−0.515 ***	−0.239 **	−0.145	0.019	0.179	−0.391 ***	−0.386 ***	−0.342 ***	0.139	
	male	−0.101	−0.703 ***	−0.570 ***	−0.213 *	0.061	−0.061	0.353 ***	−0.297 **	−0.461 ***	−0.319 ***	−0.039	
Protein (g/kg BW/day)	total	0.050	0.163 **	0.197 **	0.060	−0.006	−0.053	−0.097	0.107	0.109	0.042	−0.048	−0.314 ***
	female	0.003	0.273 ***	0.228 **	0.065	−0.116	−0.079	−0.190	0.155 *	0.097	0.065	−0.035	−0.321 ***
	male	0.060	0.216 *	0.209 *	0.030	−0.085	−0.089	0.052	−0.037	0.067	0.000	−0.012	−0.291 **

Notes: * *p* < 0.050, ** *p* < 0.010, *** *p* < 0.001.

**Table 5:** Hierarchical multiple regression results for the physical function tests and physical performance score.

PP Tests	Multiple Regression Models							
	Model	R^2^	F	∆R^2^	∆F	Ind. Variables	B	β
CST	Model 1	0.108	12.313 ***	0.099	12.313 **	Constant Age Sex BMI	20.294 *** −0.055 * 0.675 * −0.141 ***	−0.138 0.118 −0.235
	Model 2	0.142	12.607 ***	0.131	12.145 **	Constant Age Sex BMI PhA	12.827 *** −0.016 0.351 −0.132 *** 0.885 **	−0.040 0.061 −0.221 0.226
HG	Model 1	0.774	351.328 ***	0.772	351.328 ***	Constant Age Sex BMI	67.182 *** −0.571 *** 15.534 *** −0.013	−0.369 0.704 −0.005
	Model 2	0.781	272.191 ***	0.778	8.619 **	Constant Age Sex BMI Resistance	79.156 *** −0.554 *** 13.849 *** −0.160 −0.017 **	−0.358 0.628 −0.066 −0.125
	Model 3	0.784	220.890 ***	0.780	4.222 *	Constant Age Sex BMI Resistance PhA	69.060 *** −0.508 *** 13.702 *** −0.138 −0.015 * 1.031 *	−0.328 0.621 −0.057 −0.110 0.067
AC	Model 1	0.136	11.549 ***	0.124	11.549 ***	Constant Age Sex BMI	30.298 *** −0.188 *** 1.977 *** −0.030	−0.240 0.261 −0.034
	Model 2	0.155	10.110 ***	0.140	5.146 *	Constant Age Sex BMI Resistance	40.573 *** −0.200 *** 0.993 −0.151 * −0.012 *	−0.255 0.131 −0.174 −0.241
TUG	Model 1	0.307	32.605 ***	0.297	32.605 ***	Constant Age Sex BMI	−1.647 0.078 *** −0.668 *** 0.067 ***	0.361 −0.319 0.276
	Model 2	0.320	25.868 ***	0.308	4.228 *	Constant Age Sex BMI PhA	−0.122 0.071 *** −0.597 *** 0.065 *** −0.196 *	0.328 −0.285 0.272 −0.124
GS	Model 1	0.575	141.297 ***	0.571	141.297 ***	Constant Age Sex BMI	5.625 *** −0.040 *** 0.390 *** −0.029 ***	−0.500 0.347 −0.244
	Model 2	0.585	109.820 ***	0.579	7.110 **	Constant Age Sex BMI PhA	44.851 *** −0.036 *** 0.358 *** −0.028 *** 0.091 **	−0.449 0.318 −0.237 0.118
6MWT	Model 1	0.682	224.011 ***	0.679	224.011 ***	Constant Age Sex BMI	1597.978 *** −11.329 *** 81.936 *** −8.965 ***	−0.592 0.296 −0.309
	Model 2	0.701	183.220 ***	0.698	20.016 ***	Constant Age Sex BMI PhA	1322.378 *** −9.826 *** 71.023 *** −8.684 *** 32.042 ***	−0.513 0.257 −0.299 0.170
	Model 3	0.714	155.229 ***	0.709	13.621 ***	Constant Age Sex BMI PhA Resistance	1443.476 *** −9.849 *** 40.679 ** −11.397 *** 57.426 *** −3.704 ***	−0.514 0.147 −0.392 0.305 −0.206
PP score	Model 1	0.471	92.123 ***	0.466	92.123 ***	Constant Age Sex BMI	21.221 *** −0.219 *** −1.003 *** −0.159 ***	−0.630 −0.204 −0.295
	Model 2	0.500	77.334 ***	0.494	17.901 ***	Constant Age Sex BMI PhA	15.362 *** −0.187 *** −1.245 *** −0.156 *** 0.702 ***	−0.539 −0.254 −0.290 0.206
	Model 3	0.509	63.850 ***	0.501	5.453 *	Constant Age Sex BMI PhA Reactance	17.116 *** −0.187 *** −1.687 *** −0.196 *** 1.076 *** −0.054 *	−0.539 −0.344 −0.363 0.316 −0.166

Notes: PP = physical performance; R^2^ = coefficient of determination; F = F statistic; ∆R^2^ = adjusted R^2^; ∆F = changes in F; B = unstandardized regression coefficient; β = standardized coefficient; BMI = body mass index; PhA = phase angle; CST = 30 s chair stand; HG = handgrip strength; AC = 30 s arm curl; TUG = timed up and go; GS = gait speed; 6MWT = 6 min walk test; PPscore = physical performance score; * *p* < 0.050, ** *p* < 0.010, *** *p* < 0.001.

## Data Availability

The data presented in this study are available on request from the corresponding author.
